# Polypropylene/Graphene Nanocomposites: Effects of GNP Loading and Compatibilizers on the Mechanical and Thermal Properties

**DOI:** 10.3390/ma12233924

**Published:** 2019-11-27

**Authors:** Mohammad A. Al-Saleh, Abdirahman A. Yussuf, Salah Al-Enezi, Roaya Kazemi, Mat Uzir Wahit, Tahani Al-Shammari, Aseel Al-Banna

**Affiliations:** 1 Polymeric Products and Customization Program, Petroleum Research Center, Kuwait Institute for Scientific Research, P.O. Box: 24885, Safat 13109, Kuwait; ayussuf@kisr.edu.kw (A.A.Y.); senezi@kisr.edu.kw (S.A.-E.); rkazemi@kisr.edu.kw (R.K.); tshamary@kisr.edu.kw (T.A.-S.); abanna@kisr.edu.kw (A.A.-B.); 2 Center for Advanced Composites Material, School of Chemical and Energy Engineering, Faculty of Engineering, Universiti Teknologi Malaysia, Skudai Johor Bahru 81310, Malaysia; r-uzir@utm.my

**Keywords:** nanocomposite, graphene, compatibilizer, polypropylene, mechanical and thermal properties

## Abstract

In this research work, graphene nanoplatelets (GNP) were selected as alternative reinforcing nanofillers to enhance the properties of polypropylene (PP) using different compatibilizers called polypropylene grafted maleic anhydride (PP-g-MA) and ethylene-octene elastomer grafted maleic anhydride (POE-g-MA). A twin screw extruder was used to compound PP, GNP, and either the PP-g-MA or POE-g-MA compatibilizer. The effect of GNP loading on mechanical and thermal properties of neat PP was investigated. Furthermore, the influence and performance of different compatibilizers on the final properties, such as mechanical and thermal, were discussed and reported. Tensile, flexural, impact, melting temperature, crystallization temperature, and thermal stability were evaluated by using a universal testing system, differential scanning calorimetry (DSC), and thermogravimetric analysis (TGA). For mechanical properties, it was found that increasing GNP content from 1 wt.% to 5 wt.% increased tensile strength of the neat PP up to 4 MPa. The influence of compatibilizers on the mechanical properties had been discussed and reported. For instance, the addition of PP-g-MA compatibilizer improved tensile strength of neat PP with GNP loading. However, the addition of compatibilizer POE-g-MA slightly decreased the tensile strength of neat PP. A similar trend of behavior was observed for flexural strength. For thermal properties, it was found that both GNP loading and compatibilizers have no significant influence on both crystallization and melting temperature of neat PP. For thermal stability, however, it was found that increasing the GNP loading had a significant influence on improving the thermal behavior of neat PP. Furthermore, the addition of compatibilizers into the PP/GNP nanocomposite had slightly improved the thermal stability of neat PP.

## 1. Introduction

Graphene as a material has attracted a great deal of attention in material science due to its ability to improve mechanical, thermal, and electrical properties of the polymer nanocomposite. Recently, this has encouraged significant research work and interest from both academia and industry [1,2,3,4]. Furthermore, considerable research efforts have been made on the polymer blend using different nanofillers for improving mechanical and thermal properties [3,5,6]. In the literature, it has been reported that using graphene nanoplatelets (GNP) as nanofiller have enhanced the final properties of the neat polymer. For instance, polyethylene terephthalate (PET) has been studied in References [7,8,9] to improve its final properties. It was found that increasing GNP content improved the mechanical and thermal properties. When Wang et al. [10] have studied the synergistic effect of GNP and carbon nanotube using high density polyethylene (HDPE) as a base polymer, it was found that the electrical properties improved. However, a slight decrease of mechanical properties was observed. It was also reported that the improvement in mechanical and electrical properties of graphene-based polymer nanocomposites is much better than that of nano-clay or other carbon filler-based polymer nanocomposites [1,5]. Others have reported the influence of GNP on the mechanical and morphological properties of GNP reinforced PET/PP blend using a melt blending technique [11].

Polypropylene is a large commodity product used for many applications due to its low cost and its processability. Therefore, numerous research studies on PP nanocomposites have been reported in the literature using GNP as a reinforcing nanofiller [12,13,14,15]. Overall, it was found that increasing GNP loading improves mechanical and thermal properties. It is generally known that the addition of nanofiller into the polymer matrix could further improve the required properties such as mechanical and thermal properties. However, limited work has been done to study the effects and influence of compatibilizers on the final properties of PP/GNP nanocomposites, particularly comparing the performance of different compatibilizers. 

Hence, the main focus and objective of this research was to prepare the PP/GNP nanocomposite by incorporating PP-g-MA and POE-g-MA compatibilizers using the melt compounding method, and investigate the influence of GNP loading and the compatibilizers on the mechanical and thermal properties. 

## 2. Experimental

### 2.1. Materials

Polypropylene homopolymer (TITANPRO@ 6331) was used as a base polymer and was supplied by Lotte Chemical Titan (M) Sdn. Bhd (Kuala Lumpur, Malaysia). The melt flow index and density of PP are 14 g/10 min and 900 kg/m^3^, respectively. The compatibilizers (PP-g-MA (NG2002) and POE-g-MA (Fusabond@N493)) were supplied by Shanghai Jianqiao Plastic Co., Ltd. (Shanghai, China) and Dupont (Midland, MI, USA) respectively. Exfoliated graphite nanoplatelets, GNP-M-5 grade (99.5% carbon) consist of graphene nanoplatelets with an average of 5 μm in diameter and a thickness of 6 nm in a dry powder form was supplied by XG Sciences, Inc. (Lansing, MI, USA).

### 2.2. Sample Preparation and Compounding

The nanocomposites were melt compounded according to the composition shown in Table 1 using a twin screw extruder (Werner and Pfleiderer Compounding Extruder ZSK 300, Dinkelsbühl, Germany). Polypropylene pellets were dried at 40 °C overnight prior to compounding in an air-circulated oven to remove moisture. The extruder was operated at a temperature of 180 °C (feeder), 190 °C (zone 2), 200 °C (zone 3), 210 °C (zone 4), 200 °C (zone 5), and 190 °C (die) and a screw speed of 27 rpm was used during processing. The pelletized samples were then injection molded using (HTW58 Haitian, China) injection molding machine in order to prepare the test specimens. 

## 3. Characterization

### 3.1. Mechanical Properties

#### 3.1.1. Tensile

The tensile tests were performed according to ASTM D638 [16] using the Instron 5984 model. The tensile properties known as tensile strength and elongation at break were recorded. The rate used was 50 mm/min with 1 kN load. The thickness of the sample was measured using vernier callipers. Five specimens were tested and the average of the five best measurements was reported. All tests were done under room temperature.

#### 3.1.2. Flexural

The flexural tests were performed according to ASTM D790 [17] using the Instron 5984 model. The procedure used was test method 1 (three-point loading utilizing center loading) with a span width of 50 mm. The flexural properties known as flexural strength and flexural modulus were recorded. The rate used was 3 mm/min with 150 kN load. The thickness of the sample was measured using calipers. Five specimens were tested and the average of the five best measurements were reported. All tests were done under room temperature.

#### 3.1.3. Impact Testing

The impact strength of the materials was measured by using Instron Ceast Charpy. Unnotched samples with a span length of 80 mm were found, according to ASTM D6110 [18]. All specimens had the following dimensions: 127 × 12.7 × 6.35 mm^3^. For each kind of blend, five specimens were tested and the average value is given. All tests were done under room temperature. 

### 3.2. Thermal Properties

#### 3.2.1. Differential Scanning Calorimetry

Differential scanning calorimeter (DSC) measurements were carried out using a DSC-60A Plus, manufactured by Shimadzu in Japan. The starting temperature was 25 °C and was raised to 300 °C. Then it was cooled down to room temperature under a nitrogen atmosphere at a heating and cooling rate of 10 °C min^−1^. Thermal properties, such as melting enthalpy ($∆H_{m})$, melting temperature (T_m_), crystallization temperature (T_c_), and degree of crystallinity (X_c_), were calculated from DSC traces and recorded. The degree of crystallinity (X_c_) of the samples was calculated from the melting enthalpy ($∆H_{m})$ results for each sample using Equation (1).

(1) $$\%\;\mathrm{Crystallinity}\;\left(X_{c}\right)=100\times \frac{∆H_{m}}{f\times ∆H_{m}^{{}^{\circ}}},$$

where $∆H_{m}$ is the measured melting enthalpy, f is the weight fraction of the PP phase, and $∆H_{m}^{{}^{\circ}}$ is the enthalpy of 100% crystalline PP, which is 209 J/g [19].

#### 3.2.2. Thermogravimetric Analysis

Thermogravimetric analysis (TGA) was performed by using a TA Discovery Series instrument, manufactured by TA (New Castle, DE, USA). The sample was heated from 25 °C to 700 °C at a heating rate of 10 °C min^−1^ under nitrogen atmosphere. TGA parameters, such as onset temperature, were directly calculated from TGA traces. It corresponds to the temperature at which the weight loss begins.

## 4. Results and Discussions

### 4.1. Tensile Strength 

The influence of graphene nanoplatelets (GNP), with compatibilizer PP-g-MA and POE-g-MA loading on tensile strength, and elongation at break are shown in Table 2. As shown in Table 2, the tensile strength increases with an increasing GNP loading, without the compatibilizer (sample ID*), from 16.5 MPa for neat PP to 20.18 MPa for 5 wt.% GNP loading, with an increment of about 4 MPa. This increase and improvement of tensile strength by increasing GNP content could be attributed to the uniform dispersion of the nanoplatelets within the polymer matrix, which, as a result causes affective stress transfer between the polymer matrix and the nanofiller [15,20]. As shown in Table 2, a significant decrease in elongation at break was observed with increasing GNP content in the polymer matrix. For instance, the percentage elongation decreased from 88% for neat PP to 21% for 5 wt.% GNP loading. This drastic decrease in elongation could attribute high restriction in the chain movement within the polymer matrix due to higher loading of GNP filled in the system, which eventually causes stiffness in the nanocomposite. This phenomena is consistent with previously reported work in the literature [15].

Generally, the tensile strength of nanocomposites depends on the number factors such as compatibility between a polar and a nonpolar compound in the polymer blend. Therefore, as shown in Table 2, it can be seen that tensile strength of virgin PP loaded with GNP are increased further with the addition of the PP-g-MA compatibilizer when compared to PP loaded with GNP without the compatibilizer, which causes lack of compatibility between GNP and the PP matrix. As shown in Table 2, Samples (sample ID**), with PP-g-MA, exhibited the overall maximum tensile strength. This improvement of tensile strength is attributed to the existence of the compatibilizer PP-g-MA, which causes wetting of the surface interface between the polymer matrix and the graphene nanoplatelets. Hence, it improves the tensile strength of the compound. This shows the critical role of the compatibilizer. Similar observations reported in the literature [11] were the addition of compatibilizer SEBS-g-MAH, which has improved the tensile strength of the PET/PP nanocomposite. Meanwhile, the addition of POE-g-MA compatibilizer (sample ID***) into nanocomposites decreases tensile strength. As shown in Table 2, it is clearly observed that the compatibilizer PP-g-MA had better performance compared to the other compatibilizer (POE-g-MA), which indicated PP-g-MA had stronger reactivity towards PP compared to POE-g-MA, and eventually achieved better interaction between PP and GNP nanoplatelets in the polymer matrix. Hence, it can be concluded that the compatibilization of PP/GNP nanocomposite blends utilizing POE-g-MA could be insufficient to achieve full compatibility of PP/GNP nanocomposite, and, as a result, POE-g-MA tends to promote less adherence between both phases in the blend nanocomposite, and reduces the efficiency of the compatible agent, which could mean that it cannot act as an interfacial compatibilizer for the PP/GNP nanocomposite. As shown in Table 2, a significant further decrease in elongation at break was observed for both compatibilizers with increasing GNP content in the polymer matrix, and this decrease is more pronounced with the POE-g-MA compatibilizer. 

### 4.2. Flexural and Impact 

Table 3 shows the influence of GNP and compatibilizers on flexural strength and impact strength of virgin PP. The flexural strength increases slightly with increasing GNP loading (sample ID*), without the compatibilizer, from 38.85 MPa for neat PP to 40 MPa for 5 wt.% GNP loading, with an increment of about 2 MPa. As shown in Table 3, it can be seen that the addition of the PP-g-MA compatibilizer (sample ID**) into the compound has no significant improvement in flexural strength, while the addition of the POE-g-MA compatibilizer (sample ID***) has decreased the flexural strength from 38.85 MPa to 33 MPa for neat PP and 5 wt.% GNP loading, respectively. This decrease attributed less interaction between PP and GNP nanoplatelets for this particular compatibilizer (POE-g-MA), as discussed earlier in tensile strength results.

In terms of impact strength, as shown in Table 3 (sample ID*) for samples with only GNP and no compatibilizer, a reduction in impact strength was clearly observed with greater GNP loading when compared to the neat PP. This decrease in impact strength could be attributed to incompatibility between the polymer matrix and nanofiller. It is worth mentioning that further addition of 5 wt.% GNP into PP slightly increased impact strength. This could mean that, at 4 wt.%, GNP corresponds to the optimum nanofiller critical concentration in which maximum uniform dispersion can be achieved for GNP. During further addition of GNP into the polymer matrix, a graphene restacking phenomenon occurs due to van der Waals attraction within the nanoplatelets, which will have a detrimental effect on the mechanical property improvement. As shown in Table 3, the addition of PP-g-MA and POE-g-MA compatibilizers into PP/GNP increases the impact strength when compared to the PP/GNP compound without the compatibilizer. This improvement in impact strength indicates the increase of composite ductility and toughness due to the enhanced adhesion between PP and GNP phases. However, it is noted that using the POE-g-MA compatibilizer has achieved higher impact strength of the PP/GNP nanocomposite. This could be due to the existence of different adhesion effects for these compatibilizers compared to PP-g-MA, which means POE-g-MA will act as an impact modifier. A similar trend of behavior was observed by Zhang et al. [21].

### 4.3. Differential Scanning Calorimetry

**Figure 1:**
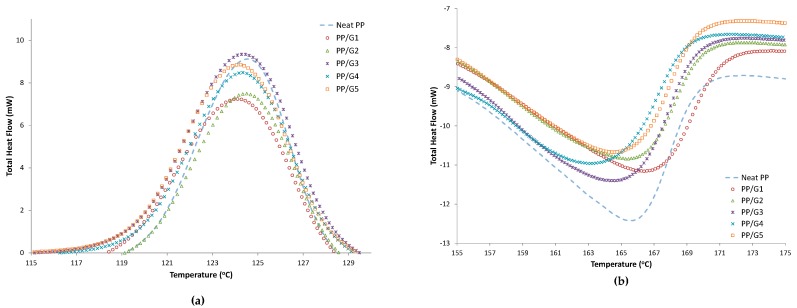
DSC cooling (**a**) and heating curves (**b**) for PP/GNP samples without compatibilizers (Sample ID*).

**Figure 2:**
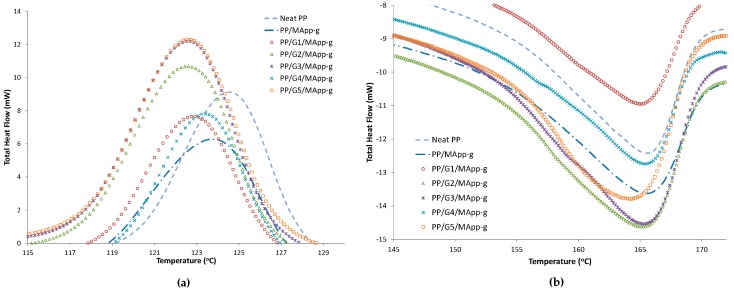
DSC cooling (**a**) and heating curves (**b**) curves for PP/GNP/PP-g-MA (Sample ID**).

**Figure 3:**
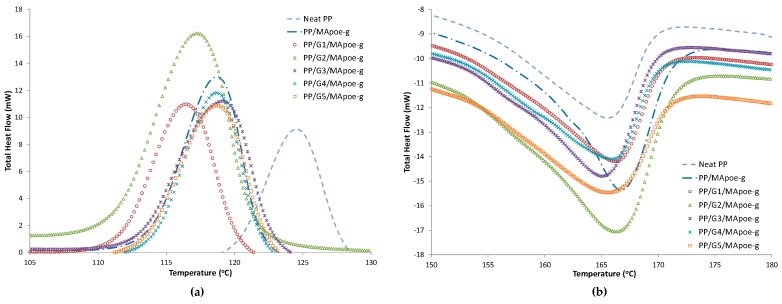
DSC cooling (**a**) and heating curves (**b**) curves for PP/GNP/POE-g-MA (Sample ID***).

The thermal properties of the PP nanocomposites were evaluated as a function of graphene and compatibilizer loading. Figure 1, Figure 2 and Figure 3 show the DSC cooling and heating curves for PP/GNP, PP/GNP/PP-g-MA, and PP/GNP/POE-g-MA samples, respectively. All results are tabulated in Table 4, which presents the crystallization temperature (T_C_), the melting temperature (T_m_), and the percentage of crystallinity (X_c_) obtained from the DSC analysis results for all samples. As shown in Figure 1 and Table 3, increasing GNP loading has no significant influence on both crystallization and the melting temperature of PP. This means that the crystal size of PP had no change that could influence the melting temperature for higher GNP content. This could be attributed to the weak nucleation effects of the nanoplatelets for PP, and the tendency of the nanofiller to suppress crystal growth in the PP matrix, which limits the formation of more crystals [22]. However, a slight increase of the percent crystallinity of PP was observed with increasing GNP loading.

Similarly, as shown in Figure 2 and Table 3, the addition of compatibilizer (PP-g-MA) to the blend had no significant influence on crystallization and melting temperature. This could indicate that the blend reaches the saturation point at lower GNP loading, and could be a disruptive effect that GNP loading has on the PP chain by limiting the freedom of the polymer chain movement. However, a slight increase of percent crystallinity was observed.

For compatibilizer POE-g-MA (sample ID***), as shown in Figure 3 and Table 4, no significant change in melting temperature and percent crystallinity were observed. However, crystallization temperature was dropped 124.59 °C to 118.64 °C. This decrease of crystallization temperature for this compatibilizer could be attributed if it was not effective in the nucleating crystal, and, as previously mentioned, GNP hinders polymer chain mobility, which limits the formation of more crystals. It can be seen that addition of this compatibilizer can slow down and decelerate the crystallization of PP.

### 4.4. Thermogravimetric Analysis 

**Figure 4:**
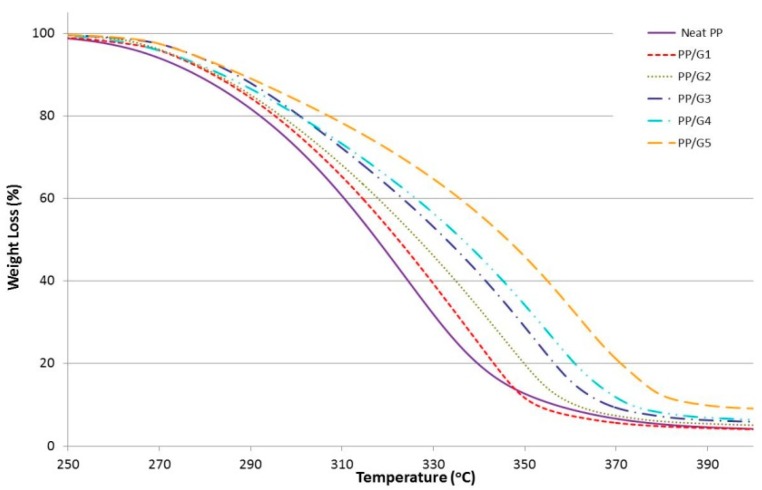
TGA curves for the first set of graphene nanocomposites without compatibilizers.

**Figure 5:**
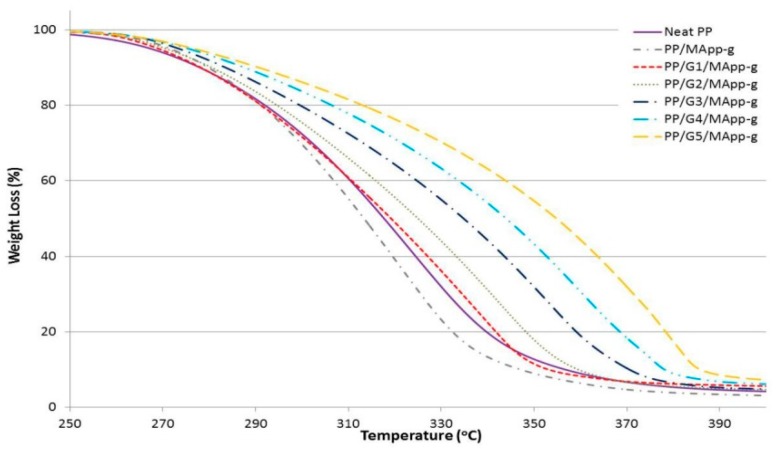
TGA curves for the second set of graphene nanocomposites with PP-g-MA.

**Figure 6:**
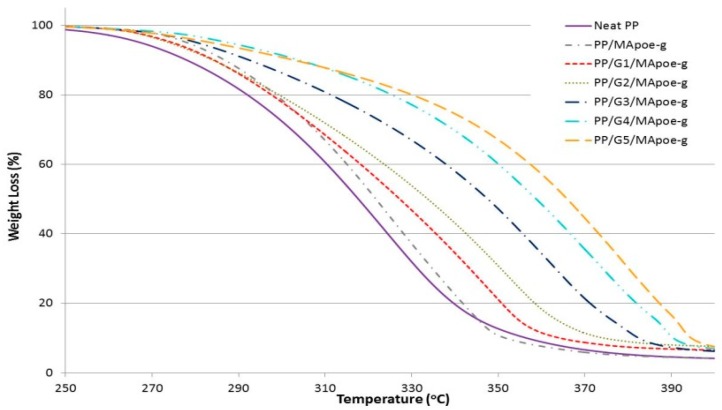
TGA curves for the third set of graphene nanocomposites with POE-g-MA.

Thermal stability for all samples were investigated and studied with TGA analysis. All the results are shown in Figure 4, Figure 5 and Figure 6 and tabulated in Table 5. The onset temperatures (T_20%_ and T_50%_) of 20% and 50% weight loss, respectively, were used as the indicator of the sample’s thermal stability. As shown in Figure 4 and Table 5, increasing the GNP loading has a significant influence on improving the thermal behavior of the nanocomposite, and showed higher thermal stability compared to neat PP. For instance, the onset temperature (T_20%_ and T_50%_) values of neat PP were about 291 °C and 317 °C, respectively, while that of 5 wt.% GNP content were 306 °C and 346 °C, respectively. It is clearly shown that the incorporation of GNP into the PP matrix significantly improved initial thermal stability of PP. This is due to the prevention of the oxygen from the material, where GNP is acting as a barrier and the insulator improves the thermal resistance of the material and, as a result, increases the thermal stability of neat PP.

As shown in Figure 5 and Table 5, in terms of samples with compatibilizer PP-g-MA, a slight decrease of the thermal stability was observed for both T_20%_ and T_50%_, particularly at the GNP filler content of 1 wt.% to 3 wt.%. However, slight improvement of thermal stability of the samples was observed at 3 wt.% and 4 wt.% GNP loading. This indicates that the compatibilizer PP-g-MA has a significant influence on thermal stability of PP at higher content of GNP, and less effect on a lower level of GNP loading, which could mean that, at lower GNP content, there were no significant improvement on thermal properties of PP by using this compatibilizer.

However, compatibilizer POE-g-MA has shown slight thermal improvement for neat PP. For instance, as shown in Figure 6 and Table 5, the onset temperatures (T_20%_) and (T_50%_) values of neat PP occurs at 291 °C and 317 °C, respectively, while that from 1 wt.% to 5 wt.% of GNP loading grew were from 296 °C to 306 °C, and 322 °C to 346 °C for T_20%_ and T_50%_, respectively. On the other hand, a slight increase of thermal stability was observed for samples containing the compatibilizer POE-g-MA. For instance, the onset temperatures (T_20%_) and (T_50%_) the thermal stability temperature occurs at 296 and 321 °C, while that of 1 wt.% to 5 wt.% of GNP loading were from 297 °C to 329 °C, and 327 °C to 366 °C for T_20%_ and T_50%_, respectively. This compatibilizer performs slightly better than the other in thermal stability improvement. A Similar trend of behavior was observed in impact strength results, and could be due to the existence of different adhesion effects for these compatibilizers, as mentioned in the impact strength results for similar samples. 

## 5. Conclusions

Polypropylene/GNP nanocomposites compatibilized with different compatibilizers, PP-g-MA and POE-g-MA, were successfully prepared by melt compounding using a twin screw extruder. The right selection of compatibilizer for polymer/GNP compounding is an important issue. Therefore, in this work, the influence and performance of compatibilizers on PP/GNP has been studied by means of mechanical and thermal tests. In addition, mechanical and thermal properties of PP and its nanocomposites were investigated. It was found that the tensile strength of neat PP increased by about 4 MPa by increasing GNP loading up to 5 wt.%. However, a significant decrease in elongation at break was observed with increasing GNP content in the PP. For the addition of the compatibilizer, it was noted that the tensile strength of virgin PP loaded with GNP are increased further with the addition of the PP-g-MA compatibilizer when compared to PP loaded with GNP without the compatibilizer. However, the addition of the POE-g-MA compatibilizer into neat PP with GNP loading decreases tensile strength. A significant further decrease in elongation at break was observed for both compatibilizers with increasing GNP content in the PP matrix, and this decrease is more pronounced with the POE-g-MA compatibilizer. It is clearly observed that the compatibilizer PP-g-MA had better performance when compared to the other compatibilizer (POE-g-MA), which indicates PP-g-MA had stronger reactivity toward PP compared to POE-g-MA. The flexural strength increases slightly with increasing GNP loading, without the compatibilizer, from 38.85 MPa for neat PP to 40 MPa for 5 wt.% GNP loading, with an increment of about 2 MPa. It can be seen that the addition of the PP-g-MA compatibilizer into the compound has no significant improvement in flexural strength while the addition of the POE-g-MA compatibilizer has decreased the flexural strength of virgin PP. For impact strength, the addition of PP-g-MA and POE-g-MA compatibilizers into PP/GNP increases the impact strength compared to the PP/GNP compound without the compatibilizer, which indicates the increase of composite ductility and toughness due to the enhanced adhesion between PP and GNP phases. However, it is noted that using POE-g-MA as a compatibilizer has achieved higher impact strength of the PP/GNP nanocomposite. For thermal analysis, it was found that increasing GNP loading has no significant influence on both crystallization and melting temperature of neat PP. However, a slight increase on the percent crystallinity of PP was observed with greater GNP loading. Similarly, it was found that the addition of compatibilizers to the PP/GNP blend have no significant influence on crystallization and the melting temperature. For thermal degradation, it was found that increasing the GNP loading has a significant influence on improving the thermal behavior of the nanocomposite. This showed higher thermal stability compared to neat PP. However, a slight improvement of thermal stability of the samples was observed at 3 wt.% and 4 wt.% GNP loading for compatibilizer PP-g-MA. Slightly thermal stability improvement for neat PP was observed by using compatibilizer POE-g-MA. 

The effectiveness of the compatibilizer is mainly controlled by its chemical structure, processing conditions, and reactive group concentration. Therefore, its mechanism is very complex and needs further studies. Lastly, it could be concluded and recommended that further studies on a detailed compatibilization mechanism are needed to understand the synergetic effects between GNP and different compatibilizers.

## Figures and Tables

**Table 1:** Material designation and composition.

Sample Designation	PP (wt.%)	Graphene (wt.%)	PP-g-MA (wt.%)	POE-g-MA (wt.%)
PP	100	-	-	-
PP/G1	99	1	-	-
PP/G2	98	2	-	-
PP/G3	97	3	-	-
PP/G4	96	4	-	-
PP/G5	95	5	-	-
PP/MA_pp-g_	96	-	4	-
PP/G1/MA_pp-g_	95	1	4	-
PP/G2/MA_pp-g_	94	2	4	-
PP/G3/MA_pp-g_	93	3	4	-
PP/G4/MA_pp-g_	92	4	4	-
PP/G5/MA_pp-g_	91	5	4	-
PP/MA_poe-g_	96	-	-	4
PP/G1/MA_poe-g_	95	1	-	4
PP/G2/MA_poe-g_	94	2	-	4
PP/G3/MA_poe-g_	93	3	-	4
PP/G4/MA_poe-g_	92	4	-	4
PP/G5/MA_poe-g_	91	5	-	4

PP: Polypropylene, G: Graphene, MA_pp-g_: PP-g-MA, MA_Poe-g_: POE-g-MA.

**Table 2:** Effects of GNP and compatibilizers on tensile strength and elongation at break.

Sample ID*	Tensile Strength (MPa)	Elongation (%)	Sample ID**	Tensile Strength (MPa)	Elongation (%)	Sample ID***	Tensile Strength (MPa)	Elongation (%)
Neat PP	16.05 ± 0.37	88.24 ± 6.39	PP/MA_pp-g_	16.57 ± 0.58	58.56 ± 6.89	PP/MA_poe-g_	15.48 ± 0.21	66.10 ± 0.40
PP/G1	18.87 ± 0.60	29.81 ± 3.42	PP/G1/MA_pp-g_	20.22 ± 0.75	29.54 ± 2.53	PP/G1/MA_poe-g_	15.10 ± 0.81	32.67 ± 0.16
PP/G2	19.44 ± 0.69	26.87 ± 6.44	PP/G2/MA_pp-g_	21.39 ± 0.38	22.65 ± 0.52	PP/G2/MA_poe-g_	17.19 ± 0.28	29.37 ± 0.59
PP/G3	19.67 ± 1.08	26.94 ± 3.72	PP/G3/MA_pp-g_	21.54 ± 0.27	18.45 ± 0.33	PP/G3/MA_poe-g_	18.45 ± 0.47	30.79 ± 0.66
PP/G4	20.08 ± 0.59	24.90 ± 7.12	PP/G4/MA_pp-g_	21.78 ± 0.50	15.55 ± 2.22	PP/G4/MA_poe-g_	18.00 ± 0.58	29.05 ± 0.71
PP/G5	20.18 ± 0.30	20.66 ± 3.00	PP/G5/MA_pp-g_	21.80 ± 0.21	16.63 ± 1.45	PP/G5/MA_poe-g_	18.73 ± 0.12	17.97 ± 0.52

Sample ID*: GNP only, Sample ID**: GNP with PP-g-MA, Sample ID***: GNP with POE-g-MA.

**Table 3:** Effects of GNP loading and the compatibilizer on flexural strength and impact strength.

Sample ID*	Flexural Strength (MPa)	Impact Strength (J\m)	Sample ID**	Flexural Strength (MPa)	Impact Strength (J\m)	Sample ID***	Flexural Strength (MPa)	Impact Strength (J\m)
Neat PP	38.85 ± 0.37	974.64 ± 23.92	PP/MA_pp-g_	40.77 ± 0.76	933.59 ± 2.61	PP/MA_poe-g_	31.72 ± 0.31	898.60 ± 14.01
PP/G1	39.06 ± 0.45	716.67 ± 65.50	PP/G1/MA_pp-g_	38.64 ± 0.6	869.03 ± 43.00	PP/G1/MA_poe-g_	32.78 ± 0.23	893.93 ± 11.59
PP/G2	39.01 ± 1.11	649.27 ± 10.76	PP/G2/MA_pp-g_	38.60 ± 0.34	702.01 ± 4.70	PP/G2/MA_poe-g_	32.57 ± 0.88	895.17 ± 2.93
PP/G3	39.74 ± 1.04	444.24 ± 37.86	PP/G3/MA_pp-g_	38.88 ± 0.54	532.90 ± 10.92	PP/G3/MA_poe-g_	32.49 ± 0.32	950.98 ± 33.31
PP/G4	40.02 ± 1.58	320.54 ± 16.67	PP/G4/MA_pp-g_	39.12 ± 0.95	384.14 ± 46.01	PP/G4/MA_poe-g_	31.68 ± 1.30	868.12 ± 3.45
PP/G5	40.05 ± 0.20	423.10 ± 36.11	PP/G5/MA_pp-g_	39.42 ± 0.48	519.87 ± 56.69	PP/G5/MA_poe-g_	33.39 ± 0.35	480.74 ± 51.38

Sample ID*: GNP only, Sample ID**: GNP with PP-g-MA, Sample ID***: GNP with POE-g-MA.

**Table 4:** Overall DSC results for all samples.

Sample ID*	T_c_ (°C)	T_m_ (°C)	X_C_ (%)	Sample ID**	T_c_ (°C)	T_m_ (°C)	X_C_ (%)	Sample ID***	T_c_ (°C)	T_m_ (°C)	X_c_ (%)
Neat PP	124.59	165.63	30.81	PP/MA_pp-g_	123.72	165.43	32.09	PP/MA_poe-g_	118.64	166.80	29.94
PP/G1	124.11	166.39	32.81	PP/G1/MA_pp-g_	122.92	164.96	39.73	PP/G1/MA_poe-g_	116.46	166.24	32.41
PP/G2	124.51	165.27	27.14	PP/G2/MA_pp-g_	122.61	165.13	42.51	PP/G2/MA_poe-g_	117.33	166.29	31.64
PP/G3	124.28	164.51	36.04	PP/G3/MA_pp-g_	122.51	165.31	33.18	PP/G3/MA_poe-g_	119.19	165.06	32.15
PP/G4	124.34	163.14	33.93	PP/G4/MA_pp-g_	123.41	165.40	27.31	PP/G4/MA_poe-g_	118.70	165.94	30.94
PP/G5	124.24	164.59	30.77	PP/G5/MA_pp-g_	122.55	164.14	31.65	PP/G5/MA_poe-g_	118.66	165.54	27.42

* The effect of GNP weight has been deducted. T_c_: crystallization temperature, T_m_: melting temperature, and X_c_: crystallinity.

**Table 5:** Overall TGA results for all samples.

Sample ID*	Weight Loss (T_20%_), °C	Weight Loss (T_50%_), °C	Sample ID**	Weight Loss (T_20%_), °C	Weight Loss (T_50%_), °C	Sample ID***	Weight Loss (T_20%_), °C	Weight Loss (T_50%_), °C
Neat PP	291	317.00	PP/MA_pp-g_	290	313.00	PP/MA_poe-g_	296	321.00
PP/G1	296	322.00	PP/G1/MA_pp-g_	291	319.00	PP/G1/MA_poe-g_	297	327.00
PP/G2	297	326.00	PP/G2/MA_pp-g_	294	325.00	PP/G2/MA_poe-g_	299	333.00
PP/G3	299	332.00	PP/G3/MA_pp-g_	299	334.00	PP/G3/MA_poe-g_	311	347.00
PP/G4	300	336.00	PP/G4/MA_pp-g_	306	343.00	PP/G4/MA_poe-g_	325	358.00
PP/G5	306	346.00	PP/G5/MA_pp-g_	313	354.00	PP/G5/MA_poe-g_	329	366.00

Sample ID*: GNP only, Sample ID**: GNP with PP-g-MA, Sample ID***: GNP with POE-g-MA.
